# Distinct differentiation characteristics of individual human embryonic stem cell lines

**DOI:** 10.1186/1471-213X-6-40

**Published:** 2006-08-08

**Authors:** Milla Mikkola, Cia Olsson, Jaan Palgi, Jarkko Ustinov, Tiina Palomaki, Nina Horelli-Kuitunen, Sakari Knuutila, Karolina Lundin, Timo Otonkoski, Timo Tuuri

**Affiliations:** 1 Program of Developmental and Reproductive Biology, Biomedicum Helsinki, PO Box 63, 00014 University of Helsinki, Helsinki, Finland; 2 Family Federation of Finland, Infertility Clinic, Fredrikinkatu 47, 00100 Helsinki, Finland; 3 Medix Laboratories Inc, Helsinki, Finland; 4 Laboratory of Cytomolecular Genetics, Department of Pathology, Haartman Institute and HUSLAB, University of Helsinki and Helsinki University Central Hospital, 00029 Helsinki, Finland; 5 Hospital for Children and Adolescents, Helsinki University Central Hospital, 00029 Helsinki, Finland

## Abstract

**Background:**

Individual differences between human embryonic stem cell (hESC) lines are poorly understood. Here, we describe the derivation of five hESC lines (called FES 21, 22, 29, 30 and 61) from frozen-thawed human embryos and compare their individual differentiation characteristic.

**Results:**

The cell lines were cultured either on human or mouse feeder cells. The cells grew significantly faster and could be passaged enzymatically only on mouse feeders. However, this was found to lead to chromosomal instability after prolonged culture. All hESC lines expressed the established markers of pluripotent cells as well as several primordial germ cell (PGC) marker genes in a uniform manner. However, the cell lines showed distinct features in their spontaneous differentiation patterns. The embryoid body (EB) formation frequency of FES 30 cell line was significantly lower than that of other lines and cells within the EBs differentiated less readily. Likewise, teratomas derived from FES 30 cells were constantly cystic and showed only minor solid tissue formation with a monotonous differentiation pattern as compared with the other lines.

**Conclusion:**

hESC lines may differ substantially in their differentiation properties although they appear similar in the undifferentiated state.

## Background

Embryonic stem (ES) cells are pluripotent cells derived from the inner cell mass (ICM) of the mammalian blastocyst. They have the potential to differentiate, both *in vitro *and *in vivo*, into derivatives of all three embryonic germ cell layers. According to current understanding, ES cells can be maintained in an undifferentiated stage indefinitely in adequate culture conditions [1].

The first human ES cell (hESC) lines were isolated in 1998 raising hopes for hESC derived cell replacement therapies for various degenerative diseases [2]. As the ES cell lines are derived from individual embryos, they are likely to have unique characteristics. Recent studies focusing on epigenetic of different hESC lines have indeed indicated that individual cell lines may have distinct line specific epigenetic profiles [3,4] which may affect their differentiation properties. Adaptation to distinct cell culture conditions may cause selection pressure altering the features of the cell lines at the epigenetic or chromosomal level. While there are plenty of studies regarding differentiation of hESC into a variety of cell types, only a few studies address the differences between individual cell lines and they are mainly focused on transcriptional profiling. Although these studies have indicated that the mRNA expression patterns do vary to some extent between the hESC lines, biological significance of the variation has not been studied in detail [5-9].

Undifferentiated ES cells are characterized by a distinct morphology and by the expression of molecular markers typical for mammalian pluripotent cells. The most commonly used cell surface markers on hESC are the glycolipid antigens SSEA-3 and SSEA-4 and the keratan sulphate proteoglygans Tra 1–60 and Tra 1–81. They are also expressed in the ICM cells of human blastocysts as well as in pluripotent human embryonic carcinoma cells [10-12]. In addition to the surface markers, the expression of certain transcription factors is a hallmark of ES cells. *OCT-4 *(*POU-domain class-5 transcription factor*, *POU5f1*) is the best known and most widely used. It is expressed in the ICM of the blastocyst stage embryo and becomes downregulated upon differentiation [13,14]. Later, a homeoprotein named Nanog was found to be present exclusively in undifferentiated ES cells [15] and also shown to be crucial for the formation of pluripotent ICM cells in mouse [16]. Recent studies have indicated that genes involved in the early commitment of primordial germ cells (PGC) are expressed in undifferentiated hESCs as well [17]. Indeed, it has been postulated that the closest *in vivo *equivalents of the ES cells might be the early committed PGCs rather than primitive ectoderm cells [18,19].

In this report, we describe the derivation of five hESC lines from frozen-thawed human embryos. A universal transcriptional comparison of these lines has been reported earlier [9]. We now report of the variable differentiation characteristics between individual lines and of the expression of PGC associated genes in undifferentiated and *in vitro *differentiated cells.

## Results

### Derivation and culture of the hESC lines

Out of 323 frozen-thawed zygotes, 83 (26%) formed blastocysts. Of the 83 blastocysts, 70 ICMs were successfully isolated onto human foreskin fibroblast (HFF) or mouse embryonic fibroblast (MEF) feeder cells. Five (7%) isolated ICMs formed continuously growing hESC lines. Two of the hESC lines (FES 21 and FES 22) were initially established on MEF and three (FES 29, FES 30 and 61) on HFF feeder cells. Later all the cell lines were characterized on both feeder types. The gene expression and teratoma analysis was performed with hESCs cultured on HFFs, embryoid body (EB) formation analyses with hESC cultured on MEF.

**Figure 1:**
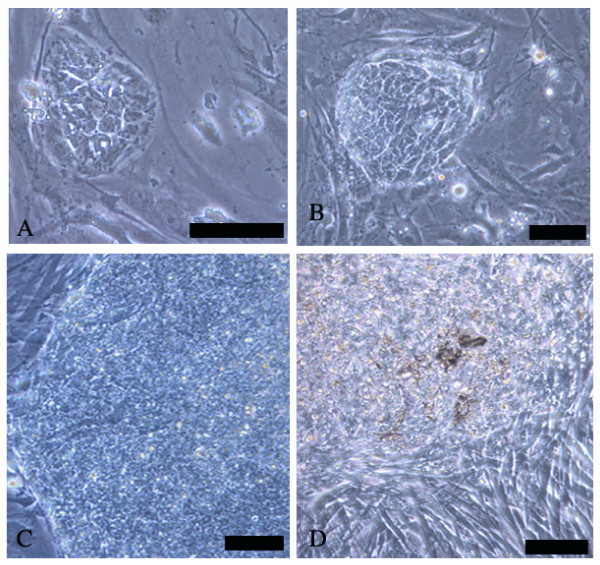
**Morphology of hESC colonies**. Cells grown on MEF (A and C) or HFF (B and D) after enzymatic passaging with collagenase IV. The initially attached colonies are smaller on MEF (A and B, FES 22 cells 36 h after passaging). At three days, the colonies on MEF (C) are larger, better defined and more homogenous than on HFF (D). Scale bar is 100 μm.

**Figure 2:**
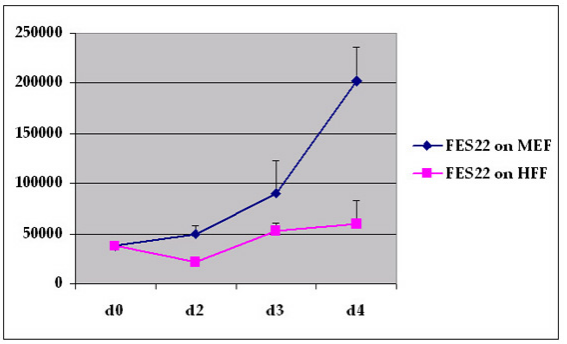
**Effect of feeders on cell proliferation**. Analysis of population doubling time on different types of feeders. Daily cell counts of triplicate wells of FES 22 cells are shown (mean ± SD).

**Figure 3:**
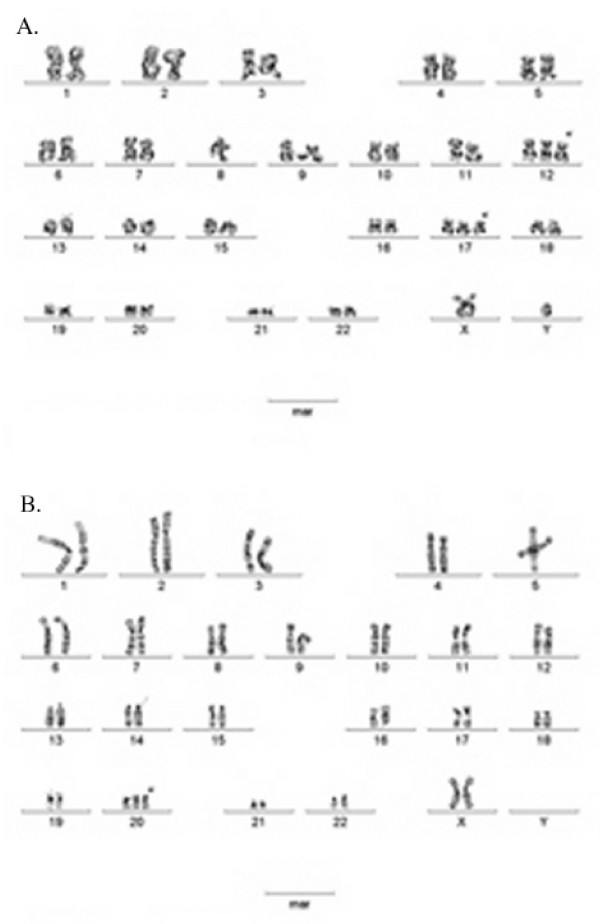
**Karyotype analysis**. Abnormal karyotypes of two FES lines. FES 22 (48, XY, +12, +17; A) and FES 30 (47, XX, +20; B) cells after prolonged enzymatic passaging on MEF.

The morphology of the hESC colonies didn't vary remarkably between the two feeder systems, if the cells were passaged mechanically. However, the density of the colonies within single dishes was clearly higher in MEF cultures than in cultures grown on HFF feeders. Notably, new hESC colonies appeared to start to grow from smaller pieces of passaged colonies on MEF than on HFF (Figure 1). While cell clusters with 20–30 cells frequently attached on MEF feeders and start to grow as undifferentiated colonies more than 100 cells/cluster appeared to be needed to initiate normal undifferentiated growth on HFF feeders. Furthermore, the small hESC clusters attached on HFF feeders differentiated more easily than on MEF feeders. We also analyzed the population doubling time of the cells in both culture systems. The average growth rate of FES 22 cells was approximately 1.5 times higher (population doubling time 24 vs. 36 h) when cultured on MEF as compared to HFF (Figure 2). The hESC lines were initially passaged mechanically by splitting colonies in up to 5 clusters (each containing 300–400 cells) and transferring pieces on new dishes. In order to more effectively expand the cell mass, enzymatic passaging with collagenase was introduced. Although both feeder types maintained the pluripotency of the hESC lines, HFF did not support long term enzymatic passaging in our hands. Eventually (within 5–10 passages) the cells lost their pluripotency and differentiated into various lineages. Four cell lines retained a normal male karyotype 46, XY and one (FES 30) a normal female karyotype 46, XX as long as the cells were cultured on human feeders. However, after about 40 passages on mouse feeders with enzymatic passaging, abnormal karyotypes were found in two lines. The karyotype of FES 22 cells was transformed into 48, XY, +12, +17 and that of FES 30 cells into 47, XX, +20 (Figure 3).

### The expression of hESC markers

All the FES lines expressed *OCT-4 *and *NANOG *as detected by RT-PCR. The cells were also positive for the hESC associated epitopes TRA 1–60, TRA 1–81 and SSEA-4 as detected by immunohistochemistry (IHC) or flow cytometry and had strong alkaline phosphatase activity (not shown).

**Figure 4:**
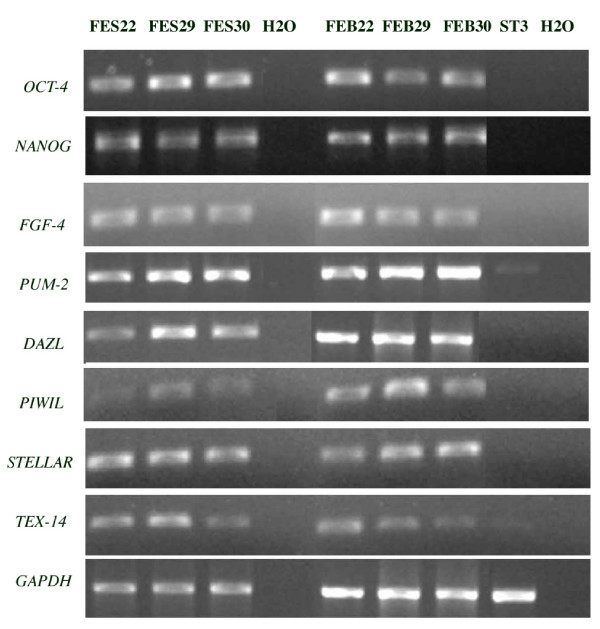
**Gene expression**. RT-PCR analysis of expression of typical genes associated with pluripotency in hESC, together with some primordial germ cell markers in undifferentiated hESCs (FES 22, 29 and 30), embryoid bodies (FEB22, 29 and 30), pooled stage 3 (ST3) differentiated cells and negative control (H_2_O).

We next identified the mRNA expression of various germ cell associated genes known or suspected to have roles in preventing the differentiation of primordial germ cells in other organisms or whose expression has previously been found to be restricted to germ cells. Their specificity for undifferentiated FES 22, 29 and 30 cells was evaluated by comparing their expression levels in undifferentiated hESCs, EBs and stage 3 (ST 3) differentiated cells (Figure 4). All the studied germ cell associated genes *DAZL*, *PUM2*, *STELLAR*, *PIWIL2 *and *TEX-14 *as well as *OCT-4*, *NANOG *and *FGF-4*, genes that have been previously described to play essential roles in the regulation of hESC pluripotency, were detected in the lines analyzed. They were also expressed in EBs, indicating either presence of a substantial amount of undifferentiated cells in EBs or that the expression of these genes is not strictly restricted to pluripotent hESCs. The more fully differentiated stage 3 cells (a mix of four cell lines) showed weak expression of *PUM2 *and *TEX-14 *transcripts, while *OCT-4*, *NANOG*, *FGF4*, *DAZL*, *STELLAR *and *PIWIL2 *were all undetectable at this stage.

### Embryoid body formation

**Figure 5:**
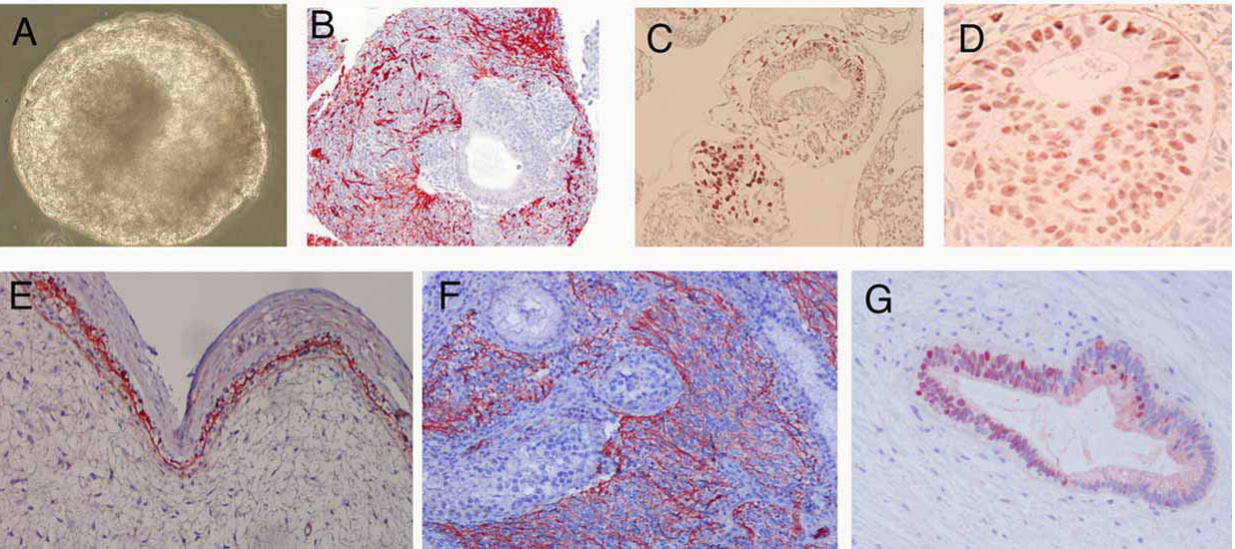
**Analysis of embryoid bodies (EBs) and teratomas**. Specific markers of all three germ cell layer derivatives detected in EBs (A-D) and teratomas (E-G) from FES line 29. Phase contrast image of EB (A). Neuroectoderm differentiation is indicated by immunostaining for neurofilament (B, F); mesoderm differentiation by expression of brachyury (C) and desmin (E); endoderm differentiation by positivity for HNF3β (D, G). Original magnification 10× (A), 20× (B, C, E-G), 40× (D).

The spontaneous differentiation capacity of the FES cell lines was evaluated both *in vitro *and *in vivo*. All the cell lines formed EBs with typical cystic morphology. The EBs contained cells expressing markers of all three germ cell layer derivatives (Figure 5A–D; staining shown for FES 29).

**Figure 6:**
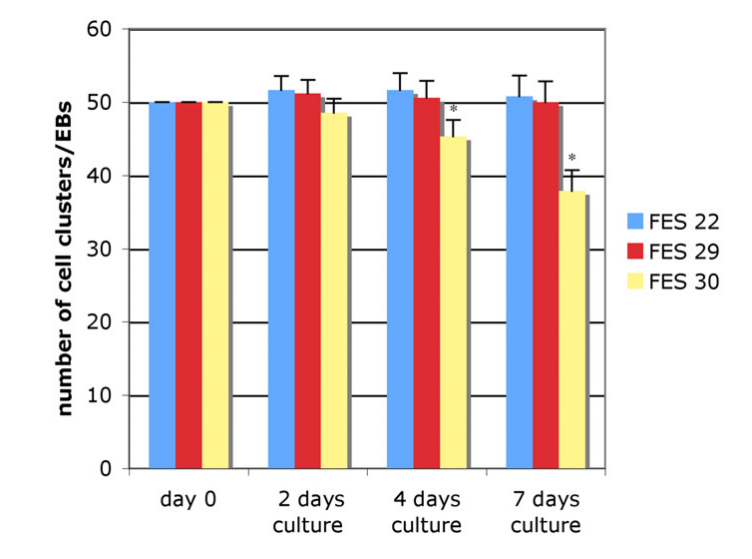
**Analysis of EB formation**. Fifty hESC colonies were placed in suspension culture and the number of developing EBs counted after 2, 4 and 7 days. The FES 30 line formed fewer EBs already after 4 days as compared to FES 22 and 29 (p < 0.01 at d 4 and < 0.001 at d 7, mean ± SD, n = 7).

**Figure 7:**
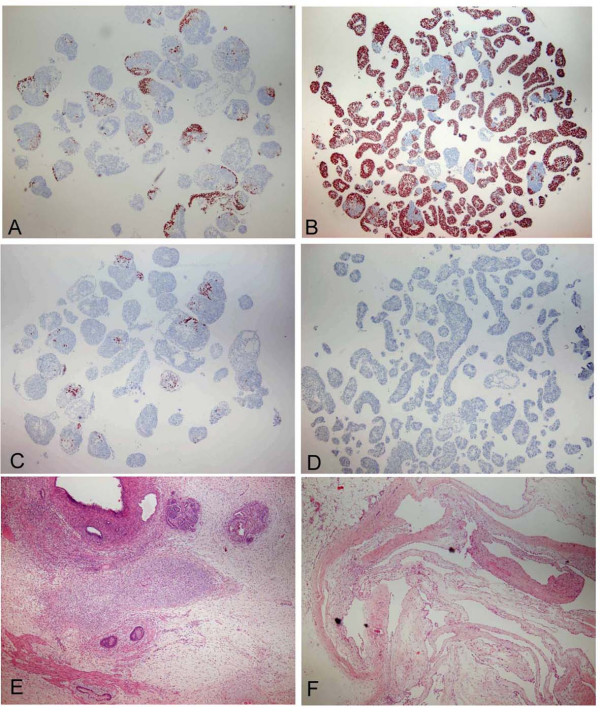
**Cell-line dependent differentiation patterns**. Spontaneous differentiation is lower in 7-day EBs (A-D) and teratomas (E-F) derived from FES 30 cells (B, D, F) than FE2 22 cells (A, C, E). OCT-4 was detected by immostaining in EBs (A-B, dark brown color) and it remained much higher in FES 30 EBs. In contrast, brachyury expression, as a marker of mesodermal differentiation, was clearly detectable in FES 22 (C) but was not detected in FES 30 EBs (D). Teratomas derived from FES 22 cells showed typical multi-lineage tissue differentiation (E) while a major part of FES 30 teratomas consisted of cystic structures (collapsed cyst wall shown in F). Original magnification 4×.

Interestingly, clear cell-line dependent variation in EB formation was reproducibly detected. The FES 30 cell line formed fewer EBs as compared to FES 22 and 29 lines (37 vs. 51 and 50 EBs per 50 colonies after 7 days, respectively) (Figure 6). We next analyzed the expression of various differentiation markers after EB formation by IHC. The total area of *OCT-4 *positive cells counted from two independent sections of approximately 50–60 EBs was clearly larger in FES 30 derived EBs (approximately 80%) than in those derived from FES 22 or 29 lines (less than 7% and about 20%, respectively, Figure 7A–B) indicating significant difference in the loss of pluripotent cells between the analyzed hESC lines. On the other hand, there was practically no expression of the mesodermal marker Brachyury in FES 30, while it was readily detectable in FES 22 and 29 derived EBs (Figure 7C–D). Similarly, FES 30 EBs showed minimal expression of endodermal HNF3β protein while the corresponding expression levels were 6–50 fold higher in EBs derived from FES 29 and FES 22 (0.08% of total section area *vs*. 0.5% and 4%, data not shown)). Altogether, the results show that in the given EB assay, FES 30 cells differentiate less readily than do FES 22 and 29 cells.

### Teratoma formation

To study the *in vivo *differentiation potential, cells from all FES lines were transplanted into nude mouse testis. Again, the differentiation patterns of the teratomas showed clear line specific features. FES 30-derived teratomas always consisted of more cystic components with a smaller solid tumor mass as compared with the other lines (FES 21, FES22, FES29 AND FES 61) (Figure 7E–F). Specific markers of all three germ layer derivatives (Figure 5E–G) were detected from teratomas of all lines both with IHC (Table 1) and RT-PCR (Table 2). Vimentin-positive mesenchymal tissues as well as desmin-positive muscle cells were abundant signs of mesodermal differentiation. Likewise, ectoderm-derived neurofilament-positive cells were frequent in most tumors, although least in FES 30 teratomas (Table 1). Definitive endodermal derivatives, characterized by gut-like mucoid epithelial structures with FoxA2/HNF-3β-positive cells were relatively abundant in teratomas derived from particularly the FES 21 line, but only rarely detected in FES 30-teratomas (Table 1). In RT-PCR analyses teratomas from FES 30 were positive for ectodermal and mesodermal marker genes but not for endodermal genes. In contrast, teratomas from other analyzed FES lines showed clear signals also for endodermal markers (Table 2).

**Table 1:** Analysis of 16 mouse intratesticular teratomas derived from the five FES lines

**Structure**	**Antigen**	**FES 21**	**FES 22**	**FES 29**	**FES 30**	**FES 61**
		n = 3	n = 4	n = 4	n = 4	n = 1

Mesenchyme (Mesoderm)	Vimentin	++++	++++	+++	+++	++++
Muscle (Mesoderm)	Desmin	+++	+++	+	+	++
Epithelium	CK-19	+++	++	++	++	++
Neuron (Ectoderm)	Neurofilament	++	++	++	+	++
Endoderm	HNF-3β	++	+	+	+/-	++
Cell proliferation	Ki-67	++	++	++	+	++
Gut-like structures		+++	+++	++	+/-	+++
Cystic tumor		-	-	+/-	++	-

**Table 2:** Gene expression in teratomas of FES lines 22, 29, 30 and 61.

**Germ layer**	**Marker gene**	**FES 22**	**FES29**	**FES61**	**FES30**
Mesoderm (muscle)	*C-ACTIN*	+	+	+	+
Mesoderm (muscle)	*MYOSIN*	+	+	+	+
Mesoderm (bone)	*CMP*	+	+	+	+
Ectoderm (neuron)	*NEUROFILAMENT L*	+	+	+	-
Ectoderm (neuron)	*TUBULIN 3β*	+	+	+	+
Endoderm	*FOXA2 *(*HNF-3β*)	+	+	+	-
Endoderm	*HNF1a*	+	+	+	-
Endoderm	*HNF1b*	+	+	+	-
Endoderm	*HNF6*	+	+	+	-
Endoderm (liver)	*ALBUMIN*	+	+	+	-
Endoderm (pancreas)	*PDX-1*	+	+	+	-
ES cell	*OCT-4*	-	-	-	-

## Discussion

We have successfully derived and expanded five hESC lines characterized by previously established criteria, including the capacity to differentiate both *in vitro *and *in vivo *into derivatives of all three germ cell layers. However, distinct line specific features were observed in terms of differentiation patterns. One of the novel lines, FES 30, repeatedly produced fewer, less diverse and smaller EBs than the other lines. FES 30 cells also maintained the expression of pluripotency-associated genes longer and differentiated less readily than the other lines when allowed to form embryoid bodies *in vitro *or teratomas *in vivo*.

In our previous genetic profiling of the four FES lines [9] the overall expression patterns were remarkably similar and FES 30 cells did not differ from the others in any major way. The expression levels of known pluripotency factors *OCT-4 *and *NANOG *that might be involved in the regulation of the differentiation tendency of hESC were verified by qRT-PCR revealing fairly constant expression of these genes in all cell lines. Yet, "functional tests" like teratoma or EB formation indicate that prominent variation between the cell lines may exist. Parallel to our finding, a recent study with mouse embryonic stem cell (mESC) indicated that even monozygotic twin mESC lines (i.e. two individual lines derived from a single ICM by dissecting the ICM into two parts prior to establishing the cell line) have differences in their differentiation patterns, suggesting that individual cells/cell compartments within the ICM may be committed to distinct developmental pathways or that at the time of ESC line formation the microenvironment may direct ESC line properties [20]. Several other hypothetical reasons for variable differentiation capacities can be postulated. For example some lines may be more prone to general or regional epigenetic changes occurring during culture and this might affect differentiation properties of the hESC lines. Alternatively, the embryos from which the lines are derived may have developmental defects that are not evident at blastocyst stage but manifest only later. Interestingly, the FES 30 cell line is the only female cell line used in our study. The role of the gender is expected to be irrelevant in early developmental processes but it can be hypothesized that for example epigenetic processes could be differently controlled in female and male lines which might also affect differentiation potential of the hESC. Nevertheless, this finding highlights the importance of conducting biological experiments with more than one cell line before drawing general conclusions about hESC physiology.

Three of the novel hESC lines were established on human foreskin fibroblasts as reported by Hovatta et al [21] in order to minimize the exposure of the cells to non-human compounds. Only five hESC lines could be established out of the 70 successful ICM isolations, which correspond to a 7% success rate. In our IVF clinic the respective implantation rate (= fetuses/transferred embryos) for blastocysts derived from frozen-thawed embryos is over 30%, indicating that the quality of the embryos should probably allow a substantially higher success rate for hESC line establishment. Based on our experience, HFFs do not support hESC growth as well as MEFs. However, it has been shown [22] that there is substantial variation between different human feeder types in their capacity to support the self-renewal of hESCs. It is possible that another human feeder cell type could support hESC growth and self-renewal better than our current HFFs. This may also have affected the derivation efficacy of new hESC lines.

All the FES lines have been in continuous culture for one to three years (50–120 passages) without losing their pluripotency. Normal 46 XX (FES 30) or XY (FES 21, 29, 30 and 61) karyotypes have been detected at least twice for every FES line, mainly on HFF feeders. However, after transferring the cells on MEF feeders and starting to passage them enzymatically, changes in the chromosomal constitutions of FES 22 and 30 involving chromosomes 12, 17 and 20 were found. Similar kinds of changes (recurrent gain of chrs 17q and 12) have been reported for three other hESC lines [23]. Cowan et al [24] reported the derivation of 17 new hESC cell lines that were passaged by trypsin from the beginning. General chromosomal changes (trisomy, additions to chr 2) were observed in these lines after prolonged culture. One possible explanation for this may be linked to the passaging method. Enzymatic trypsin- or collagenase-based methods most likely favor the selection of fast growing cells, which may have undergone mutations or chromosomal rearrangements. We propose that mechanical splitting methods should be used initially for the first 20–30 passages to ensure that karyotypically normal frozen stocks can be established. These findings also emphasize the importance of frequent karyotype analyses to assure that results obtained in any studies with hESCs are not biased because of abnormal chromosomal constitution.

To identify new specific markers for hESC cells and to reveal genes possibly involved in the maintenance of pluripotency, we analyzed the mRNA expression of several germ cell associated genes at various stages of hESC differentiation. The fact that hESCs (and mESCs) express several markers that distinguish epiblast cells from early PGCs as well as some other findings related to the formation of mESC lines have raised the question whether ES cells might actually be more closely related to PGCs than epiblastic cells [19]. *Stella *(also known as *Dppa3 *or *Pgc7*) is a transcription factor exclusively expressed in a subset of mouse epiblastic cells committed to become primordial germ cells [25]. *DAZL *is an autosomal homologue of the Y-chromosomal *DAZ *gene and is expressed in humans also in oocytes and preimplantation embryos up to blastocyst stage [26]. Recently, *DAZ *and *DAZL *were shown to interact with PUM2 protein [27] which is a homologue to *Drosophila pumilio*, an essential gene in maintaining the normal primordial germ cell population of the adult fly [28]. According to our expression analyses, these genes, as well as testes specific *PIWIL2 *and *TEX-14 *[29,30], can be used as markers for undifferentiated hESCs. The exact biological roles or interactions with other genes of any of these PGC/germ cell associated genes are not known. However, our results indicate that hESCs express several germ cell associated genes that may be involved in the molecular mechanisms regulating early differentiation and/or self-renewal of these cells.

## Conclusion

We have derived five new hESC lines and characterized them extensively. Several important lessons can be learned from this work: First, due to poorly understood reasons, the lines may vary substantially in their spontaneous differentiation properties even though there are no obvious differences in the generally accepted ES cell characteristics in the undifferentiated state. Second, in order to obtain a cell growth rate required for many purposes, the hESCs need to be grown on embryonic mouse feeders which also make it possible to use enzymatic instead of mechanical passaging of the cells. However, these conditions may of the lead to chromosomal instability. Third, several genes expressed in primordial germ cells may serve as useful additional pluripotency markers.

## Methods

### Derivation of hESCs

*In vitro *fertilized excess human embryos were donated for the generation of hESC lines after an informed consent of the respective couples. The Ethical Committee of the Hospital district of Helsinki and Uusimaa has approved the generation of hESC lines. The embryos frozen at day 2 were thawed and cultured up to blastocysts. The ICMs of day 5 or 6 blastocysts were isolated by first degrading zona pelluzida using 0.5% Pronase (Sigma-Aldrich/YA-Kemia, Helsinki, Finland). For the lines FES 21, 22, 29 and 30, the trophectoderm was removed by immunosurgery using rabbit anti-human serum (Sigma-Aldrich) and guinea pig serum complement (Sigma-Aldrich). For FES 61 the trophectoderm was removed mechanically by needles. The hESC lines FES 21 and FES 22 were initially derived and cultured on mouse embryonic fibroblast feeders (MEF; 1213 pc fetuses of the ICR strain) and FES 29, 30 and 61 cell lines on human foreskin fibroblasts feeder cells (HFF; CRL-2429 ATCC, Mananas, USA). FES 21 and 22 were cultured on MEF for 10 and 28 passages, respectively. Thereafter they were transferred on HFF.

### Culture of hESCs (stage1)

The ICMs and hESCs were cultured either on HFF or MEF feeders (mitotically inactivated by Mitomycin-C, density 27 000 cells/cm^2 ^and 10 000 cells/cm^2^, respectively) in serum-free medium (KnockoutD-MEM; Invitrogen, Paisley, UK) supplemented with 2 mM L-Glutamin/Penicillin streptomycin (Sigma-Aldrich), 20% Knockout Serum Replacement (Gibco), 1 X non-essential amino acids (Gibco), 0.1 mM betamercaptoethanol (Gibco), 1 X ITS (Sigma-Aldrich) and 4 ng/ml recombinant bFGF (Invitrogen). The first splitting after ICM isolation was done after 10–14 days by mechanical disaggregation. The growing cell aggregates were then passaged to new plates at 5–7 day intervals. HESCs cultured on HFFs were passaged mechanically while those cultured on MEFs were passaged either mechanically or enzymatically. For mechanical passaging the colonies (which approximately contained 2000 cells) were cut into 4–5 pieces, and transferred on new dishes (20–30 pieces/dish). For enzymatic passaging the cells were exposed to 200 units/ml collagenase IV (Gibco) for 5–10 min at 37°C, dissociated by gently pipetting and plated on 2–3 new dishes.

### Population doubling time

Depending on the experiment, 10 000 to 40 000 enzymatically passaged hESCs that had been cultured on MEFs were plated on 24-well tissue culture plates pre-seeded either with MEF or HFF feeder cells. After two days, three wells with MEF and HFF feeders without hESCs were trypsinized and counted to give the average number of feeders per well. Also, three wells of hESCs on both feeders were trypsinized and counted to give the reference number of hESCs per well. Thereafter, at time points 24 and 48 hours three wells with hESCs on both feeders were harvested and cell numbers were counted. The feeders were subtracted from total cell counts and the population doubling times of the hESCs calculated on both feeder types.

### Karyotyping

For karyotype analysis the hESCs cultured on HFF feeders were first transferred on feeder free culture for approximately 2 weeks. The cells were then harvested by trypsinization and metaphases were analyzed using conventional light microscope (Olympus, BX 50) and karyotypes were made using IKAROS-software designed for chromosome analysis (MetaSystems GmbH, Altlussheim, Germany). 20 metaphases were analysed for each cell line. Alternatively, cells grown on MEF feeders were treated directly with colchisine for two hours and then harvested and metaphases were analyzed.

### RNA isolation, RT-PCR and immunohistochemistry (IHC)

Total RNA was isolated from hESCs, EBs and cells grown out of the EBs (stage 3 differentiated cells) by SV Total RNA system (Promega, Madison, WI, USA). Total RNA from teratomas was isolated by NucleoSpin RNA II kit (Macherey-Nagel, Germany). Two μg of total RNA treated with RQ1 RNase-Free DNase (Promega,) was used for 20 μl of reverse transcription reaction with M-MLV Reverse Transcriptase (Promega). Nucleotides were purchased from Roche Applied Science (Penzberg, Germany) and oligo (dT)15 Primers from Promega. One μl of each RT reaction was used for PCR amplification with AmpliTaq Gold DNA polymerase (Applied Biosystems, CA, USA). Primer pairs used are listed in Table 3.

**Table 3:** PCR primers used.

**Name**	**GenBank**	**Sequence of 5'-primer**	**Sequence of 3'-primer**	**Product size**
DAZL	NM_001351	GGAGCTATGTTGTACCTCC	CCATGTAACTAGATAAGCCAG	313 bp
PIWIL2	BC025995	TCTATGGGGCCATCAAGAAG	CCATCCCGATCACCATTAAC	195 bp
TEX14	BK000998	TCCTGTTTTTGGAAGCGACT	GTGGCAGCTGAACAAAGTGA	214 bp
STELLA	AY317075	CACAAATGCTCACCGAAGAA	TTCGATTTCCCTGAGGACTG	182 bp
FGF4	NM_002007	TCACCGATGAGTGCACGTTCA	GAGGAAGTGGGTGACCTTCAT	158 bp
NANOG	AB093576	GGAAGACAAGGTCCCAGTCA	ATTGTTCCAGGTCTGGTTGC	349 bp
OCT4	XM_084899	CGTGAAGCTGGAGAAGGAGAAGCTG	AAGGGCCGCAGCTTACACATGTTC	245 bp
PUM2	AF315591	CCAACATTCCTTGGTGAG	ATCAGGACCCCAAGAAGAGG	402 bp
C-ACTIN	NM_005159	TGATATCCGCAAGGACCTGT	GCTGGAAGGTGGACAGAGAG	200 bp
MYOSIN	NM_013292	TCCATGTTCGACCAGACTCA	AAGCGGTCACACTGCGTGGT	335 bp
CMP	NM_002379	AGAGCTACAGCGTCATCGAG	TCTGACACGTCCAGCGTATC	316 bp
NF-L	NM_006158	CAAAGAGTGAAATGGCACGA	AGCGGGTGGACATCAGATAG	231 bp
TUBULIN3β	NM_006086	CATCCAGAGCAAGAACAGCA	TCGGTGAACTCCATCTCGTC	234 bp
ALBUMIN	XM_031320	GCACAATGAAGTGGGTAACC	CAGCAGTCAGCCATTTCACC	349 bp
FOXA2	NM_021784	TGCCAGGAGCACAAGCGAGG	TGTTCGTAGGCCTTGAGGTCC	290 bp
HNF1α	NM_000545	CAGGTCTTCACCTCAGACAC	GAGGCCATCTGGGTGGAGAT	263 bp
HNF1β	NM_000458	ACCTTGACGAATATCCACAGC	CTGTGACCACCATTGCAGATG	364 bp
HNF6	U96173	AGGGCAGATGGAAGAGATCA	TGGATGGACGCTTATTTTCC	377 bp
PDX1	U30329	ACCAAAGCTCACGCGTGGAA	CTCTCGGTCAAGTTCAACAT	191 bp
GAPDH	M33197	GTCTTCACCACCATGGAGAAGGCT	TGTAGCCCAGGATGCCCTTGAGGG	529 bp

Undifferentiated ES cell colonies were fixed in the culture dishes by 4% PFA for the immunocytochemical detection of the stem cell surface markers, SSEA-4, Tra 1–60 and Tra 1–81 (in dilutions 1:100). Primary antibodies were obtained from the Developmental Studies Hybridoma Bank (Iowa City, IA, USA; SSEA-4 and Tra 1–60) and Chemicon International (Temecula, CA, USA; Tra 1–81). The same antibody (SSEA-4) was used for flow cytometric analysis (FACSCalibur, Becton Dickinson, NJ, USA). Immunohistochemical studies of teratomas and EBs were done in paraffin sections. The rehydrated sections were microwave treated in 1 mM EDTA, pH 8, for 15 minutes to reveal antigenic sites, cooled at room temperature (RT) for 30 minutes and rinsed in aqua. Endogenous peroxidase was inactivated with 3% hydrogen peroxide in aqua for 10 minutes. After rinsing with PBS the sections were incubated in 4% normal goat or rabbit serum (Vector Laboratories, Burlingame, CA, USA) in PBS-0.1% Tween20 for 2 hours at RT to block nonspecific binding sites. Primary antibodies (mouse anti-vimentin and mouse anti-cytokeratin-19, DakoCytomation, Glostrup, Denmark; goat anti-human brachyury, rabbit anti-desmin and goat anti-HNF3β Santa Cruz Biotechnology, Santa Cruz, CA, USA; monoclonal mouse anti-neurofilament high antibody 13A8, a gift from Prof. Ismo Virtanen, University of Helsinki, Helsinki, Finland, Ki-67, Novocastra, Newcastle, UK) were diluted (1:5000, 1:500, 1:200, 1:3000, 1:5000 and 1:1000, respectively) in PBS containing 4% normal serum and Tween-20 and incubated overnight at +4 C. After rinsing several times with PBS, the sections were incubated with biotinylated goat anti-rabbit, rabbit anti-mouse or rabbit anti-goat secondary antibody (Zymed Laboratories, San Francisco, CA, USA) in PBS-Tween for 30 minutes at RT, rinsed in PBS and incubated with peroxidase conjugated streptavidin (Zymed Laboratories) diluted in PBS-Tween. The sections were finally developed with AEC substrate (3-amino-9-ethyl carbazole; Lab Vision Corporation, Fremont, CA, USA). After rinsing with water counterstaining was performed with Mayer's hemalum solution. Alkaline phosphatase activity in the hESC lines was demonstrated by a commercial kit (Chemicon).

### Embryoid body (stage 2) differentiation

To induce the formation of embryoid bodies (EBs) the hESC colonies were first allowed to grow for 10–14 days. Thereafter the colonies were cut in small pieces (approximately 5000 cells) and transferred on non-adherent Petri dishes (Becton Dickinson, NJ, USA) to form suspension cultures. The hESCs were then cultured for the next 10 days in suspension in standard culture medium (see above) without bFGF. The formed EBs were either fixed with 4% PFA and subjected to IHC analyses or used for total RNA isolation and RT-PCR analyses.

For the comparison of the EB formation efficacy, the hESCs on MEF feeders were treated with collagenase IV (1 mg/ml) for one hour. The floating colonies were then transferred to non-adherent Petri dishes, 50 colonies in each well (Becton Dickinson, NJ, USA). The experiments were repeated 7–8 times depending on cell line. After 7 days in standard culture medium the EBs were counted and fixed with 4% PFA for immunohistochemical analysis and morphometry by Image-Pro Plus 4.5 software (Media Cybernetics Inc.). Statistical significance of the observed differences was tested by unpaired Student's t test.

### Stage 3 differentiation

To induce further differentiation, embryoid bodies were transferred onto gelatin-coated (Sigma-Aldrich) adherent culture dishes in DMEM/F12 media (Gibco) supplemented with ITS, Fibronectin (Sigma), L-glutamine and antibiotics. The attached cells were grown for 10 days, whereafter they were harvested and total RNA was isolated.

### Teratoma formation

In order to study teratoma formation, about 200 000 morphologically good looking hESC cells were injected into nude mice testes. The resulting tumors were harvested at 8 weeks after injection. A part of the tumor was used for RNA isolation and the remaining part fixed with 10% formalin and immunohistologically examined. The animal experiments were approved by the experimental animal welfare committee of the District Government of Southern Finland.

## Authors' contributions

MM had a major role in the cell culture experiments, participated in the teratoma analysis and drafted the manuscript. CO participated in the cell culture experiments. JP and TP carried out the gene expression analysis. JU carried out immunocytochemical analyses. NH-K and SK carried out the karyotype analysis. KL participated in the gene expression and teratoma analysis. TO and TT conceived of the study, participated in its design and coordination and helped to draft the manuscript. All authors read and approved the final manuscript.
